# COVID-19 and African Immigrants in North Africa: A Hidden Pandemic in a Vulnerable Setting

**DOI:** 10.1017/dmp.2020.387

**Published:** 2020-10-19

**Authors:** Mohamed A. Daw, Abdallah H. El-Bouzedi, Mohamed O. Ahmed

**Affiliations:** Department of Medical Microbiology and Immunology, Faculty of Medicine, University of Tripoli, Tripoli, Libya; Department of Laboratory Medicine, Faculty of Biotechnology, University of Tripoli, Tripoli, Libya; Department of Microbiology and Parasitology, Faculty of Veterinary Medicine, University of Tripoli, Tripoli, Libya

**Keywords:** disease outbreaks, emergency preparedness, epidemics, epidemiologic methods

Since being declared a pandemic in March 2020, severe acute respiratory syndrome coronavirus 2 (SARS-CoV-2), which causes the disease known as *coronavirus disease* (*COVID-19*), has brought difficult situations for citizens of nations worldwide. The effects, however, may be more severe for vulnerable communities, such as immigrants, who are already in desperate situations and under deteriorating conditions. There are still very limited data on how the pandemic is impacting migrant communities. Immigrant camps foster an environment that poses a great threat to the health of their inhabitants, especially at the time of a pandemic. Overcrowding, poor sanitation, inadequate health care, and difficulty containing contagious diseases are well documented in African immigration detention centers.^1^ Furthermore, they are unlikely to take priority in a moment in which governments are mobilizing all resources to care for their citizens. Their situation is even more complicated if they are hosted in corridors plagued by war, as in North Africa.^2,3^

Northern African countries, particularly Libya, which has the longest coast in the Mediterranean basin, are known to be an important hub for African immigrants toward Europe. The ongoing armed conflict left the country split between 2 rival administrations vying for power, and no evident national policy to combat the pandemic was applicable. Currently, the reported cases of COVID-19 have increased overwhelmingly to reach over 23 000 cases (ratio 3/1000). The adverse policy environment in such a rich country has made the Libyan communities, particularly African immigrants, more vulnerable to the uncontrolled spread of the pandemic.^4,5^

The status of coronavirus infection among African immigrants is not well studied, and no published data highlight the impact of this infection on immigrants residing in North African countries. This study aimed to determine the serological prevalence of SARS-CoV-2 among African immigrants and outline the needed policy to combat the pandemic within this neglected community.

A total of 350 blood samples collected from different African immigrants residing in 2 immigrant camps in Libya was included in this study. Each sample was tested using the SARS-CoV-2 IgM/IgG Rapid Test. The test was performed according to the manufacturer’s instructions as previously described.^6^ The immigrants were mainly males who arrived from different African countries, ages between 20 and 50 years, as shown in Table 1. Of the tested individuals, 11 (3.1%) tested positive for SARS-CoV-2, ranging from 0.6%, among a younger age group (20–30 years old) and reaching up to 6.0% in a middle age group. No significant relation was found between the prevalence of SARS-CoV-2 and the country of origin of the immigrants. The epidemiological investigations in this study indicate the identification of SARS-CoV-2 infections within the camps of African immigrants. This is crucial for effective disease containment; once infected individuals are identified, they should be isolated, and their close contacts should be traced, which is difficult to apply in immigrant camps.

**TABLE 1:** The Prevalence of SARS-CoV-2 in African Immigrants and Its Correlation With Demographic Factors

Demographic Characteristics		Prevalence of SARS-CoV-2	
	Total	No. (%)	OR (95% CI)
**Age group**			
20–30	147	1 (0.7)	0.8 (0.6–1.3)
31–40	153	7 (4.6)	5.1 (2.1–9.4)
41–50	50	3 (6.0)	7.1 (4.8–10.2)
**Gender**			
Male	350	11 (3.1)	3.7 (2.9–5.4)
Female	00	00 (00)	00 (00–00)
**Location of immigrant center**			
Southwestern region*	200	7 (3.5)	3.9 (2.9–6.4)
Eastern region**	150	5 (3.3)	3.6 (2.8–6.1)
**Region of origin**			
North Africa	153	6 (3.9)	3.4 (2.9–10.2)
Central Africa	107	3 (2.8)	3.0 (2.9–9.8)
West Africa	90	2 (2.2)	2.9 (2.1–9.4)

*Notes:* *Libyan–Algerian borders; **Libyan–Egyptian borders.

The main limitation of this preliminary study is that there were still false results when applying the IgG-IgM combined antibody test kit, which should be taken into consideration. However, rapid serology tests provide a means to quickly triage suspected cases of COVID-19 infection, provided the test is highly specific for the disease, particularly where there is little or no access to molecular testing, as in the case of African immigrants.^6^ Furthermore, we were not able to trace the source of infection or to follow up on the clinical situation of the infected immigrants. Hence, further studies should be done to fill these gaps.

In conclusion, the disproportionate burden of the SARS-CoV-2/COVID-19 pandemic for African immigrants is distressing, and thus, immediate action by the international community and regional governments is urgently needed to implement proper public health measures to protect immigrants as residents and when they return to their countries.
